# An Optimized Histochemical Method to Assess Skeletal Muscle Glycogen and Lipid Stores Reveals Two Metabolically Distinct Populations of Type I Muscle Fibers

**DOI:** 10.1371/journal.pone.0077774

**Published:** 2013-10-30

**Authors:** Clara Prats, Alba Gomez-Cabello, Pernille Nordby, Jesper L. Andersen, Jørn W. Helge, Flemming Dela, Otto Baba, Thorkil Ploug

**Affiliations:** 1 Center for Healthy Aging, University of Copenhagen, Copenhagen, Denmark; 2 Department of Biomedical Sciences, University of Copenhagen, Copenhagen, Denmark; 3 Faculty of Health and Sport Science, University of Zaragoza, Zaragoza, Spain; 4 Institute of Sports Medicine, Bispebjerg Hospital and Center for Healthy Aging, University of Copenhagen, Copenhagen, Denmark; 5 Biostructural Science, Tokyo Medical and Dental University, Tokyo, Japan; INSERM/UMR 1048, France

## Abstract

Skeletal muscle energy metabolism has been a research focus of physiologists for more than a century. Yet, how the use of intramuscular carbohydrate and lipid energy stores are coordinated during different types of exercise remains a subject of debate. Controversy arises from contradicting data from numerous studies, which used different methodological approaches. Here we review the “pros and cons” of previously used histochemical methods and describe an optimized method to ensure the preservation and specificity of detection of both intramyocellular carbohydrate and lipid stores. For optimal preservation of muscle energy stores, air drying cryosections or cycles of freezing-thawing need to be avoided. Furthermore, optimization of the imaging settings in order to specifically image intracellular lipid droplets stained with oil red O or Bodipy-493/503 is shown. When co-staining lipid droplets with associated proteins, Bodipy-493/503 should be the dye of choice, since oil red O creates precipitates on the lipid droplets blocking the light. In order to increase the specificity of glycogen stain, an antibody against glycogen is used. The resulting method reveals the existence of two metabolically distinct myosin heavy chain I expressing fibers: I-1 fibers have a smaller crossectional area, a higher density of lipid droplets, and a tendency to lower glycogen content compared to I-2 fibers. Type I-2 fibers have similar lipid content than IIA. Exhaustive exercise lead to glycogen depletion in type IIA and IIX fibers, a reduction in lipid droplets density in both type I-1 and I-2 fibers, and a decrease in the size of lipid droplets exclusively in type I-1 fibers.

## Introduction

Skeletal muscle metabolism has been the subject of extensive research for more than a century. The initial interest to characterize skeletal muscle energy stores and its use during physical activity came from the field of exercise physiology. Understanding which energy stores are used as fuel during different types and durations of exercise and, how the recovery of different energy stores is regulated after exercise could help athletes improve their performance. Today we know that skeletal muscle metabolism is not only essential to maintain body posture and perform physical activity, skeletal muscle is also indispensable for maintaining metabolic body homeostasis and/or metabolic health. Obesity and reduced physical activity lead to metabolic dysregulation, which is strongly associated with skeletal muscle insulin resistance and development of metabolic syndrome and type 2 diabetes mellitus (T2DM) [1]. Consequently, the exponential rise in the prevalence of obesity and T2DM has also lead to a growing interest in skeletal muscle energy storage and metabolism.

Skeletal muscle stores lipids and carbohydrate mainly as intracellular lipid droplets (LDs) and glycogen particles, respectively. Both lipid and glycogen content can be measured by biochemical techniques; however, such techniques are most often performed on muscle homogenates, and can therefore not discriminate between intramyocellular and extramyocellular lipid and glucose stores, and do not allow for muscle fiber typing. In order to measure only intramyocellular energy stores and to differentiate between the different fiber types, histochemical methods have been extensively used. The two commonly used histochemical methods to asses skeletal muscle lipid and glycogen stores are staining of muscle cryosections with oil red O (ORO) [2] and periodic acid-schiff (PAS) [3], respectively.

ORO is a fat-soluble dye used for staining of neutral lipids and can be visualized by fluorescence or bright field microscopy. It stains neutral lipids not only in LDs but also forming part of intracellular membranes. It is thus critical that any publication that analyzes intramuscular lipid droplets by staining with ORO should present representative images of the stains. A similar problem exists when using PAS staining to measure skeletal muscle glycogen. The PAS staining is used to stain glycogen but it also stains glycoproteins and proteoglycans. However, diastase can be used to digest glycogen from the cryosection and differentiate the glycogen stain from other stained structures [3]. Glycogen preservation in the analyzed cryosections is critical and not always considered in the published studies. Fairchild and Fournier [4] clearly showed that commonly used steps in the standard protocol employed to measure muscle glycogen by PAS staining, like thawing and air drying muscle cryosections before fixation and staining, significantly reduces muscle glycogen content.

Here we review and optimize critical steps in the most commonly used protocols for histochemical analysis of skeletal muscle lipid and glycogen content: preservation of intramuscular glycogen and lipid stores during the staining procedure and specificity of the used dyes.

## Materials and Methods

### General Materials, Reagents and Solutions

The following reagents were used: ORO (Merck), 40% triethyl-phosphate (Sigma Aldrich), mouse anti-SERCA1 IgG (S1189, Sigma Aldrich), polyclonal rabbit anti-COXIV (Ab16056, Abcam), polyclonal rabbit anti-Laminin IgG (L9393, Sigma Aldrich), mouse anti-fast twitch myosin heavy chain (M4276, Sigma Aldrich), mouse anti-slow twitch myosin heavy chain (M8421, Sigma Aldrich) and, Hoechst H-33342 and Triton X-100 were also purchased from Sigma-Aldrich, Denmark. Bodipy-493/503, goat anti-rabbit IgG conjugated to Alexa Fluor 568, goat anti-mouse IgG conjugated to Alexa Fluor 488, goat anti-mouse IgM conjugated to Alexa Fluor 488, goat anti-rabbit IgG conjugated to Alexa Fluor 568 and, Zenon Alexa Fluor 568 and 488 mouse IgG labeling kits were purchased from Invitrogen, Denmark. The monoclonal mouse anti-glycogen IgM was raised by Dr. Otto Baba [5], [6], Vectashield mounting medium was purchased from Vector laboratories, US. Rabbit anti-MitoNEET IgG was kindly donated by Dr. Philipp E Scherer. As fixatives: 1) 4% Zamboni fixative (4% depolymerized paraformaldehyde supplemented with 0.15% picric acid [7]), 2) Acidic ethanol contained 10% glacial acetic acid in 60% ethanol and, 3) Formaldehyde in ethanol fixative, containing 4% formaldehyde and 90% ethanol were used. The immunobuffer (IB) used contained 0.25% BSA, 50 mM glycine, 0.033% saponin and 0.05% sodium azide in PBS. Muscle cryosections were mounted on SuperFrost glass slides (Menze-Gläser, Germany), in Vectashield mounting medium, covered with cover slips (#1.5) and sealed with transparent nail polish. Hydrophobic DAKO pen (DAKO, Denmark) was used to delineate a circle around muscle cryosections to retain incubation solutions on the sections.

### Human *Vastus Lateralis* Biopsies

*Vastus lateralis* (VL) muscle biopsies (n = 6) from young healthy subjects (age 26±1 years, body mass index of 24.4±0.7 kg⋅m^−2^ and VO_2_max of 57±2 ml of O_2_⋅(min⋅kg)^−1^) were obtained using the Bergström needle biopsy technique [8] before and after exhaustive exercise. VO_2_max and W_max_ were measured 3–10 days before the experimental day. To measure VO_2_max, subjects performed a graded exercise test on a bicycle ergometer (Ergometrix 800S,Ergo-line GmbH, Germany) starting at 80 W (3 min) followed by increments of 35 W every minute until exhaustion. During exercise, pulmonary VE, VO2 and VCO2 were measured by an online system (Oxycon Pro, Jaeger, Würzberg, Germany). *W_max_* was measured as previously described [9]. The day before the experiment, subjects were given instructions on how to prepare their dinner, a standardized meal containing 25% energy (*E*%) protein, 5 *E*% carbohydrate, and 70 *E*% fat. On the experimental day,subjects came to laboratory overnight fasted and, a biopsy was taken before exhaustive exercise. The exhaustive exercise protocol was a knee extensor exercise at 85% of *W*_max_ for the first 60 min, and then the workload was increased to 90% *W*_max_. After 90 min the workload was increased to 95% *W*_max_, after 105 min to 100% *W*_max_, and after 120 min to 105% *W*_max_ until exhaustion. A biopsy was taken right after the exercise bout. Muscle biopsies from the same study have been analyzed in previous studies [10], [11]. Muscle biopsies were embedded in O.C.T. compound and rapidly frozen by immersion into solid isopentane for at least 30 seconds. Samples were kept at −80°C until further processed. A Leica CM3050 S cryostat was used to cut 15 µm thick muscle cryosections for the different tests described. During the cutting, the cryostat’s chamber temperature was kept at −20°C and the object holder temperature at −15°C.

### Analysis of Intramyocellular Triacyl Glycerol (TG)

Muscle TG was measured biochemically in the muscle biopsies after treatment with tetraethylammoniumhydroxide, perchloric acid, and KHCO3. The liberated glycerol concentration in the supernatant was subsequently measured spectrophotometrically (Hitachi 612 Automatic Analyzer; Roche, Glostrup, Denmark).

### Ethic Statements

All subjects gave verbal and written consent to participate and the study was approved by the ethical committee of Copenhagen and Frederiksberg municipality (01-257174) and adhered to the Helsinki II Declaration.

### Tests for Optimization of IHC Staining

#### Test 1 – ORO *versus* bodipy

**Figure 1:**
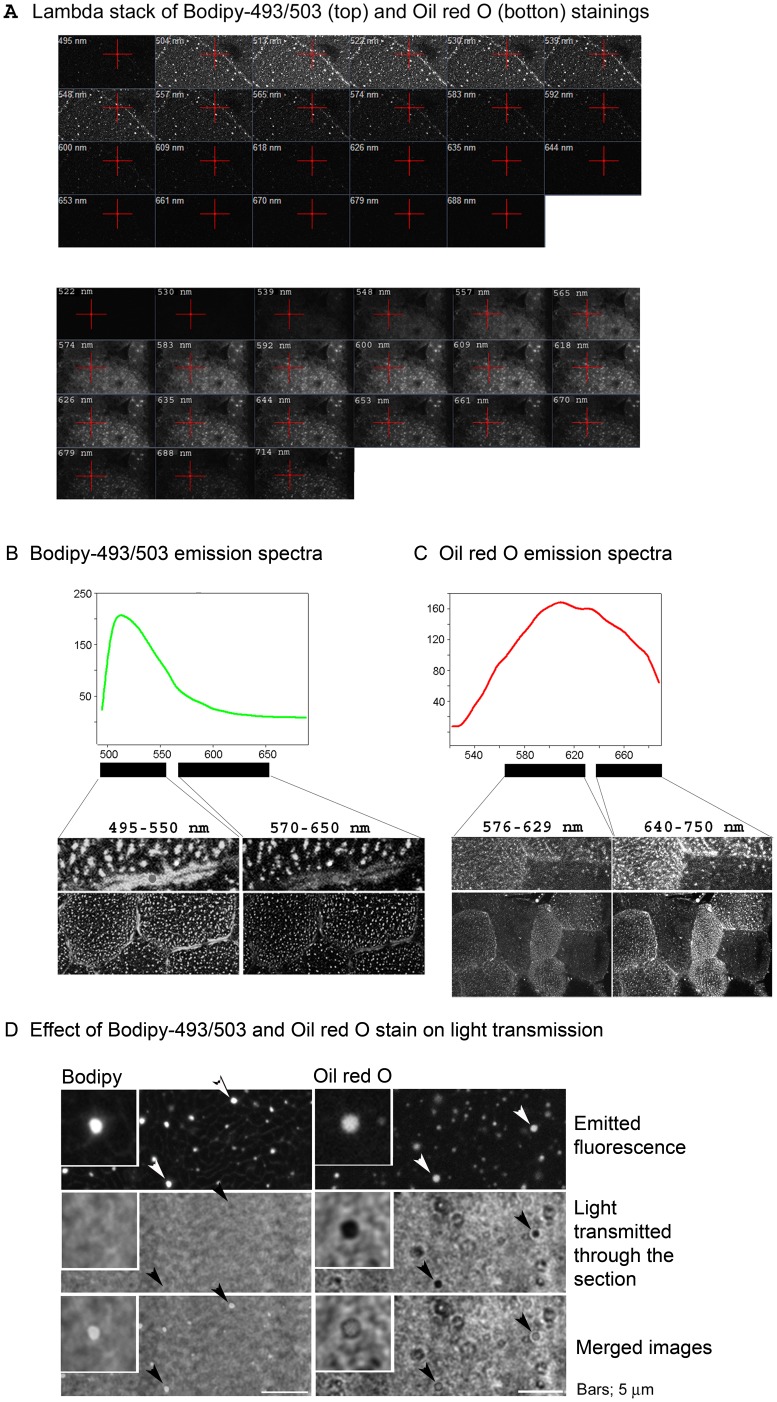
Bodipy-493/503 *versus* Oil Red O. Consecutive cryosections from human *VL* muscle were stained with ORO or Bodipy-493/503. Lambda stacks of the fluorescence emitted by Bodipy and ORO stained muscle sections were acquired with a LSM780, with a resolution of 9 nm (A). Emission spectra were graphed from the lambda stack and show that Bodipy’s emission spectra (B) is narrower than ORO’s (C). Furthermore, for both lipophilic dyes, the results show that lower emitted wave lengths seem to originate from stained intracellular membranes, while the higher wave lengths originate from the lipid droplets. (D) The detection of the emitted fluorescence from the Bodipy or ORO stained muscle cross-sections were combined with imaging of transmitted light through the cryosections, showing that transmission of light through the stained section is clearly blocked by the ORO stained lipid droplets, but unaltered by Bodipy stained lipid droplets (arrows).Bars; 5 µm.

Four, 15 µm thick, consecutive muscle cryosections were cut, mounted on two glass slides and, rapidly fixed by immersion into ice cold Zamboni fixative for 1 hour. Right after fixation, the two cryosections from one of the slides were washed with MilliQ water and incubated for 30 min with ORO, while the sections on the second slide were washed with PBS and incubated for 30 min with Bodipy. Subsequently, the cryosections stained with Bodipy (20 µg/ml in PBS) or ORO (3.3 mg ORO in 1 ml 40% triethyl-phosphate) were washed in PBS or MilliQ water, respectively, and mounted in Vectashield. Spectral imaging was performed using the spectral detector in the Zeiss LSM780, ORO being excited with a HeNe 543 nm laser line, while Bodipy was excited with a 488 nm argon laser. The emission spectra of ORO and Bodipy were graphed (Figure 1 A and B).

#### Test 2 – Air drying muscle cryosections has severe adverse effects on IMTG content and intracellular morphologic preservation

Twelve consecutive cryosections from each muscle biopsy (n = 4) were mounted on 6 different slides (two cryosections per slide). Slide 1 and 4 were rapidly immersed for 1 hour into ice cold Zamboni fixative. The cryosections mounted on slide 2 and 5 were air dried at room temperature for 15 min and, slides 3 and 6 were air dried at room temperature for 30 min before immersion fixation for 1 hour. After fixation, muscle cryosections were washed 3 times 5 min with PBS. Slides 1, 3 and 5 were incubated for 30 minutes with Bodipy and then washed with PBS before being mounted. Slides 2, 4 and 6 were incubated for 2 hours with rabbit anti-MitoNEET and mouse anti-SERCA1 diluted in IB, washed with IB 3 times 15 min and then incubated for 45 min with secondary antibodies diluted in IB (1/500). An anti-rabbit IgG conjugated to Alexa Fluor 568 (A568) and an anti-mouse IgG conjugated to Alexa Fluor 488 (A488) were used. In order to visualize myonuclei, muscle cryosections were incubated for 5 min with Hoechst in IB. This was followed by two 15 min washes with IB and one 15 min wash with PBS. Right after, the immunostained muscle sections were mounted in Vectashield mounting medium. Stack of 4 confocal images, 0.35 µm apart, were used to obtain z-maximum projections and quantify the density and size of intracellular lipid droplets using ImageJ software.

#### Test 3 – Optimization of glycogen preservation and fixation

Six slides, with each 2 muscle cryosections, were prepared from 4 different muscle biopsies. From each sample, cryosections from two slides were fixed by immersion into ice cold acidic ethanol for 10 minutes. Cryosections from 2 slides were fixed by 1 hour immersion into ice cold Zamboni fixative and the third group of cryosections was immersed for 1 hour into ice cold 4% PFA in ethanol. After fixation, one of the slides from each muscle was washed 5 min with PBS and permeabilised by a 15 min incubation at room temperature with 0.2% Triton X-100 in PBS, while the other slide was incubated with PBS for 15 min as control. After been washed muscle cryosections were incubated for 2 hours with a 1∶20 dilution of the mouse anti-glycogen IgM and 1∶25 dilution of the rabbit anti-Laminin IgG in IB. After 3 washes with IB, 10 min each, cryosections were incubated for 1 hour with a 1/500 dilution of a goat anti-mouse IgM conjugated to A488 and a goat anti-rabbit IgG conjugated to A568. After incubation with the secondary antibodies, muscle cryosections were incubated for 5 min with Hoechst in IB and, thereafter washed 2 times 5 minutes with IB and one time 5 minutes with PBS.

#### Test 4 – Staining of consecutive muscle cryosections for IMTG, myosin heavy chain I and II and, glycogen and laminin

From each muscle biopsy (n = 6), six consecutive 15 µm thick muscle cryosections were cut and fixed for 1 hour in ice cold Zamboni fixative. Two sections were washed with PBS and then incubated for 30 min in Bodipy solution. After 3 washes 5 min each, sections were mounted and imaged. In order to fiber type, two muscle cryosections were washed 3 times 5 minutes in IB and incubated for 2 hours with 1/500 mouse anti-fast twitch myosin heavy chain and 1/500 mouse anti-slow twitch myosin heavy chain antibodies. Since both primary antibodies are raised in mouse, they were pre-labeled using Zenon A568 and A488 labeling kits, respectively. Cryosections were then washed 5 min with Hoechst 33342 diluted to a working solution of 2.5 µg/ml in IB and, 2 times 5 minutes with IB. Post-fixation was performed to avoid release of stained glycogen particles by 30 min immersion in Zamboni fixative. After washing with IB for 2 times 5 minutes and 1 wash of 5 min in PBS, sections were mounted and imaged. The two last sections were used for co-immunostaining of glycogen and laminin. Muscle sections were washed 5 min with PBS and permeabilised by 15 min incubation with 0.2% Triton X-100 in PBS. After been washed 2 times 5 min muscle cryosections were incubated for 2 hours with a 1∶20 dilution of the mouse anti-glycogen IgM and 1∶25 dilution of the rabbit anti-Laminin IgG in IB. After 3 washes with IB, 10 min each, cryosections were incubated for 1 hour with a 1/500 dilution of a goat anti-mouse IgM conjugated to A488 and a goat anti-rabbit IgG conjugated to A568. Muscle cryosections were then incubated for 5 min with Hoechst in IB and, thereafter washed 2 times 5 minutes with IB and one time 5 minutes with PBS before mounting. All muscle fibers in each cryosection were analyzed, which was a mean of 163±31 and 124±18 muscle fibers per muscle biopsy in the basal and exhausted VL groups, respectively. With the obtained results, the required number of muscle fibers that need to be analyzed to obtain a representative measurement was calculated as previously described [12]. In order to get a representative sampling for glycogen content measurements, at least 20 muscle fibers of each fiber type need to be analyzed. On the other hand, in order to obtain a representative pool of muscle fibers to assess LD size and density, a minimum of 10 muscle fibers need to be analyzed per each fiber type in each biopsy.

### Confocal Image Acquisition

Image acquisition was performed with a Zeiss LSM710 through a 63x/1.40 oil DIC Plan-Apochromat objective. An argon laser 488 nm was used to excite Bodipy-493/503 and Alexa Fluor 488, while a HeNe 543 nm laser line was used to excite ORO and Alexa Fluor 568. Pinhole was kept to 1 AU and the imaging conditions set to avoid empty or saturated pixels. Imaging conditions were kept constant when acquiring images to be compared. Z-stack confocal images were collected from the top to the bottom of the cryosections and processed using Image J software (http://rsbweb.nih.gov/ij/). Spectral imaging was performed using the 32 array GaAsP spectral detector of the Zeiss LSM780 with a spectral resolution of 9 nm.

### Image Processing

Quantification of lipid and glycogen stores was performed using Image J software. A threshold was set to include lipid or glycogen signal. Thresholds were kept constant between samples. Individual muscle fibers were manually delineated and measurements performed using the multi measurement plugin. Glycogen signal was measured as mean grey value per fiber, while lipid stores were measure as number of lipid droplets per 3.6 µm^2^ and lipid droplets size as number of pixels, being a pixel 3.6*10-3 µm^2^.

### Statistics

All data is expressed as means ± SEM. Statistical evaluations were performed by: Test 2, One way repeated measures ANOVA was performed to assess the effect of air drying on IMTG staining in 4 different samples, from which at least 12 muscle fibers were analyzed. Test of freezing and thawing during the Bodipy staining protocol, Mann-Whitney Rank Sum Test was performed, since the normality test failed. Test 4, Two-way repeated measures ANOVA was performed to assess statistically significance between fiber types and before/after exhaustive exercise. Statistical significances are represented as: *vs* I-1 or Basal *vs* Exhausted: *p<0.05, **p<0.01 and ***p<0.005; *vs* I-2: #p<0.05, ##p<0.01 and ###p<0.005; *vs* IIA: ¤p<0.05, ¤¤p<0.01 and ¤¤¤p<0.001.

## Results and Discussion

### IMTG Staining in Human Muscle Cryosections - Optimization and Important Considerations

Sudan dyes have classically been used to stain sudanophilic substances, usually lipids. Most of the studies that have measured IMTG in muscle cryosections have used the Sudan dye ORO. A more recent class of lipid dyes is the Bodipy’s [13]. By flow cytometry, Bodipy-493/503 has been shown to be more specific for lipid droplets than nile red [14]. Here we investigate whether ORO or Bodipy-493/503 (Bodipy) are the best dye of choice for visualizing and quantifying IMTG in muscle cryosections. Consecutive muscle cryosections were stained with ORO or Bodipy. For exact protocol, refer to Materials & Methods (Test 1). Spectral imaging was performed (Figure 1A) and, their emission spectra plotted (Figure 1B and C). ORO and Bodipy show emission peaks at 609 nm and 513 nm, respectively. Both dyes stain intracellular neutral lipids in general and do not exclusively stain LDs. Therefore it is important to be able to discriminate between the fluorescence emitted by stained IMTGs and the fluorescence emitted by other intracellular organelles. When collecting Bodipy emitted fluorescence from 495 to 550 nm of wavelength, we observe large continuous intracellular structures, including both IMTG droplets and intracellular membranes. Interestingly, when collecting Bodipy emitted fluorescence from 570 to 650 nm, most of the collected fluorescence comes from IMTG droplets (figure 1b). Accordingly, excitation of Bodipy with a 488 nm laser and detection of the emitted fluorescence between 570 and 650 nm is a good strategy to specifically image IMTG. In contrast, staining of IMTG with ORO gives a more diffuse staining pattern, which combined with its broad emission spectrum, makes it difficult to discriminate between the fluorescence emitted by stained lipids in different intracellular compartments (figure 1c). Furthermore, when using transmission microscopy, it becomes apparent that IMTG stained with ORO are interfering with the transmitted light, while IMTG stained with Bodipy do not affect transmitted light (figure 1d). Such a characteristic is extremely important when combining IMTG staining with immunofluorescence staining of associated proteins. ORO has been used in numerous studies to investigate the association of proteins with IMTG [15]–[18]. Considering that staining of IMTG by ORO may block the light emitted by the fluorophore conjugated to the secondary antibody used to immunodetect the protein of interest, Bodipy should be the dye of choice. Therefore, bodipy is the best dye to measure IMTG and the dye of choice whenever imaging the relative intracellular distribution of IMTG and other proteins or cellular structures.

#### Air drying muscle cryosections has severe adverse effects on IMTG content and intracellular morphologic preservation

Air drying of muscle cryosections prior to staining is a routine procedure in many labs. In order to investigate the effect of air drying muscle cryosections on the preservation of IMTG and intracellular structural morphology, muscle cryosections were cut and immediately fixed by immersion into cold fixative and compared to muscle cryosections that were allowed to air dry for 15 or 30 minutes before fixation. IMTG was assessed by Bodipy staining and intracellular morphologic preservation assessed by immunofluorescence staining of mitochondria (mitoNEET) and sarcoplasmic reticulum (SERCA1). For detailed protocol of the test see Materials & Methods (test 2).

**Figure 2:**
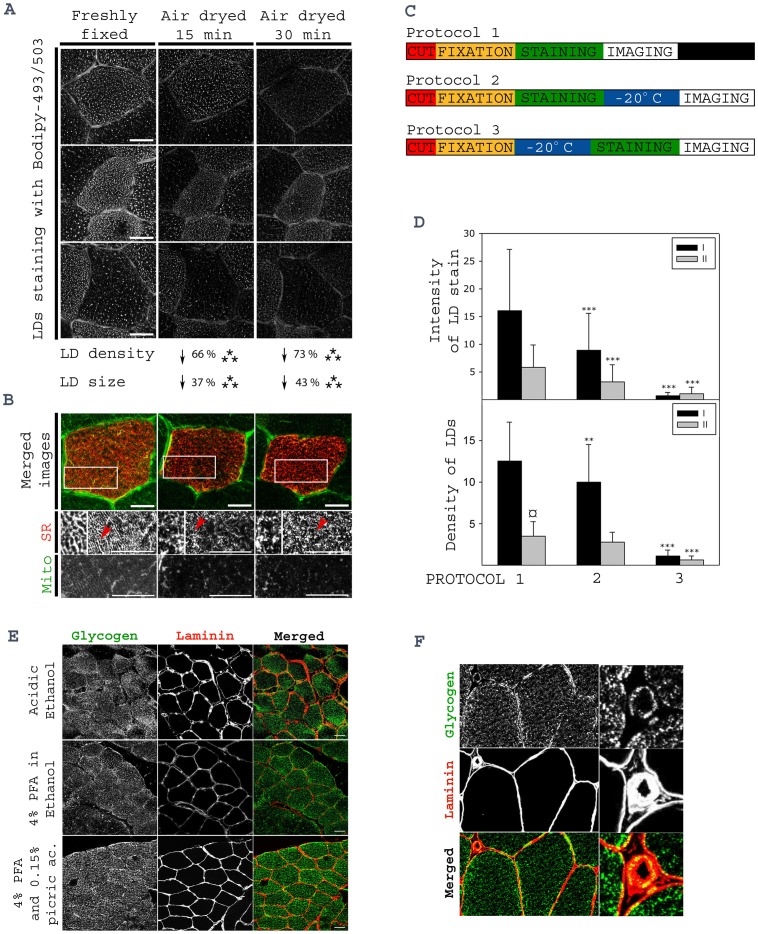
Optimization of human muscle lipid and glycogen stain. Consecutive cryosections from 4 human *VL* muscle were either cut at −20°C and fixed right away or allowed to air dry for 15 or 30 min before staining of IMTG. (A) Representative high resolution images of individual muscle fibers are shown (bars; 20 µm). For each muscle (n = 4) and each condition 12 muscle fibers were used to quantify lipid droplet size and density. Lipid droplet density decreased 66 and 73% due to 15 or 30 min air drying, respectively. Furthermore, the size of lipid droplets decreased 37 and 43% due to 15 or 30 min air drying, respectively. (B) Air drying of muscle cryosections also has an effect on the preservation of intracellular structures, such as sarcoplasmic reticulum (SR) and mitochondria (mito). Bars, 15 µm. Arrows in the representative images of SR staining point to the areas that are magnified to visualize the ultra-structural alteration of SR during air drying. (C) Three protocols were compared to investigate the effect of freezing consecutive muscle cryosections at different time points during the staining protocol. In protocol 1 muscle cryosections were cut, stained and imaged the same day. In protocol 2 and 3, muscle cryosections were frozen and kept at −20°C for 3 weeks after or before Bodipy staining, respectively. Fiber typing was performed by immunostaining against myosin heavy chain I and II on a consecutive cryosection. Quantification of lipid droplet stain intensity and lipid droplet density was performed in 20 fibers from each muscle (n = 4) and, the results are plotted (D). The intensity of lipid droplet stain is expressed in arbitrary units (mean grey value), while the density of lipid droplets is expressed as number of lipid droplets per 3.6 µm^2^. The results show a clear loss of lipid droplet staining and density in protocols 2 and 3, compared to protocol 1. Statistical significance *versus* Protocol 1 is represented as *p<0.05, **p<0.01 and ***p<0.005 and statistical significance between type I and type II is represented as ¤p<0.05 (E) In order to optimize muscle glycogen preservation in muscle cryosections, three different fixatives were tested: Acidic ethanol, 4% PFA in ethanol and 4% PFA supplemented with 0.15% picric acid (Zamboni). Representative images of glycogen and laminin co-immunostaining of cryosections fixed with the three different fixatives are shown. Bars; 20 µm. A high resolution image of glycogen and laminin co-immunostaining showing preservation of glycogen particles in arterial smooth muscle is presented (F) to highlight the optimal preservation of glycogen when using 4% PFA supplemented with 0.15% picric acid. Smooth muscle fibers are smaller and more fragile than skeletal muscle single muscle fibers.

The same muscle fibers were identified and imaged in each consecutive cryosections, so that the effect of air drying on the amount of IMTG and the structural preservation could be assessed in the same cells. A total of 12 fibers per each of the 4 muscle biopsies used were analyzed in the three tested conditions (figure 2A and B). Air drying muscle cryosections for 15 min resulted in a 66% decrease in LD density (p<0.005) and a 37% decrease in LDs’ average size (p<0.005). If the air drying time was increased to 30 min, a 73% decrease in the density of LDs (p<0.005) and a 43% decrease in the average size of the LDs (p<0.005) were observed. Furthermore, immunostaining of sarcoplasmic reticulum and mitochondrial network revealed a clear distortion of the structural preservation of both cellular organelles and, a reduction in the fibers cross sectional area after air drying for either 15 or 30 min (Figure 2 B).

#### Effect of freezing and thawing muscle cryosections before image acquisition of lipid staining

For practical reasons, it can be convenient to be able to stop the staining protocol and store the stained cryosections in order to perform the image acquisition another day. Here we tested whether muscle cryosections could be frozen and stored at −20°C before or after staining with Bodipy. Three protocols were designed (Figure 2 C). In all three protocols, two cryosections from 2 muscle biopsies were cut, mounted on a glass slide and fixed immediately by immersion for 1 hour in cold fixative. After fixation, in protocol 1, muscle cryosections were stained with Bodipy (as described above) and image acquisition performed right away. During protocol 2, muscle cryosections were also stained after fixation, but kept frozen for 3 weeks at −20°C before image acquisition. During protocol 3, muscle cryosections were stored at −20°C for 3 weeks after fixation and, thawed and stained with Bodipy before image acquisition. The intensity of IMTG stain by Bodipy and the density of LDs was assessed and showed a significant detrimental effect of freezing and thawing before or after lipid staining on IMTG preservation (figure 2D). Based on the presented data we conclude that for optimal preservation of IMTG, muscle cryosections cannot go through any freezing/thawing cycle between the cutting of the cryosection and image acquisition.

### Glycogen Staining in Human Muscle Cryosections - Optimization and Important Considerations

Periodic acid-Schiff (PAS) stain is based on the reaction of periodic acid with the diol functional groups in glucose and other sugars, oxidizing them to form aldehyde, which in turn reacts with the Schiff reagent to give a purple/magenta stain. PAS stain is thus not a glycogen specific stain; it also stains glycoproteins and proteoglycans. In order to discriminate glycogen from other PAS-reactive cellular components cryosections can be pre-treated with the glycogenolytic enzyme diastase. Although this is not optimal, most of the published studies did not perform this and glycogen content is therefore over-estimated. Furthermore, Fairchild & Fournier [4] demonstrated that thawing and air drying muscle cryosections results in glycogen degradation. Despite this, in most laboratories, where histochemical measurements of glycogen in tissue cryosections are done by PAS staining, it is common practice to thaw and dry tissue cryosections after cutting and before fixation to ensure they are firmly attached to the glass slide and flat. Here we have optimized skeletal muscle preservation and increased stain specificity by using the monoclonal anti-glycogen IgM antibody [5], [6].

#### Optimization of glycogen preservation by different fixation protocols and antibody penetration

In order to optimize glycogen preservation, muscle cryosections were cut, mounted on 6 slides and, rapidly fixed following 3 different fixation protocols: acidic ethanol, Zamboni or, 4% PFA in ethanol. After fixation, one of the two slides fixed with each protocol was permeabilised by a 15 min incubation at room temperature with 0.2% Triton X-100 in PBS, while the other slide was incubated with PBS for 15 min as control. Co-immunostaining for glycogen and laminin was performed as described in test 3, Tests for optimization of IHC staining section. Pre-incubation of muscle cryosections with Triton increased immunodetection of glycogen particles. Considering that the primary antibody against glycogen is an IgM, which is a pentamer of IgG, it is not surprising that penetration into the myofilaments of IgMs will require extra permeabilization. Representative images of the resulting staining with the three different fixation protocols are shown (Figure 2E). Optimal preservation of the amount and spatial organization of intracellular glycogen stores were obtained with Zamboni fixation. As can be seen in Figure 2F, even glycogen particles in the smooth muscle of arterioles were preserved with 1 hour fixation in Zamboni and 15 min incubation with Triton.

### Optimization of the Technique Reveals the Existence of 4 Metabolically Distinct Fiber Types in Human Skeletal Muscle

**Figure 3:**
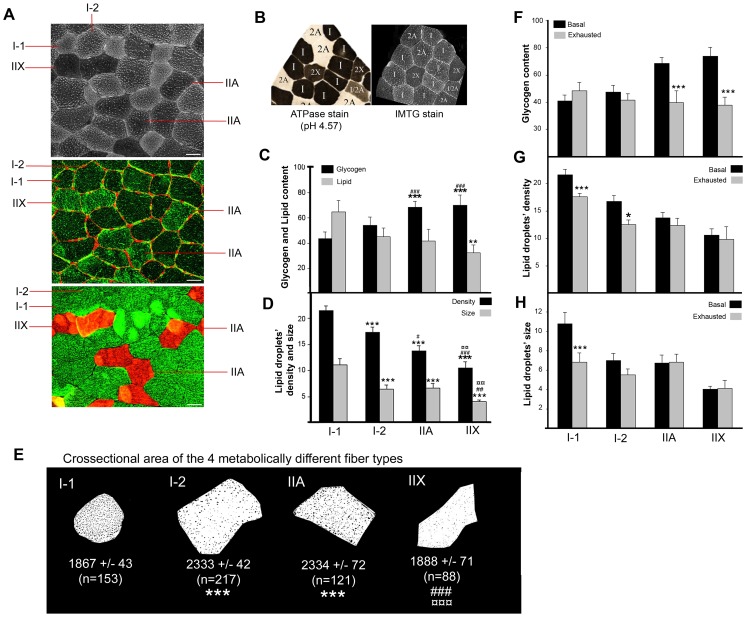
Staining of human *VL* muscle lipid and glycogen stores with an optimized protocol reveals the existence of four metabolically distinct fiber types. (A) Representative images of consecutive muscle crossections stained for lipid (top), glycogen and laminin (middle) and myosin heavy chain I (green) and II (red)(bottom) are presented. Bars; 20 µm. (B) ATPase staining of consecutive sections allowed for type IIa and IIx distinction. (C) Lipid (grey bars) and glycogen (black bars) stores were analyzed in *vastus lateteralis* muscle from 6 young healthy subjects and, the results are plotted as mean grey value ± SEM. Statistical differences *versus* I-1 are represented as *, *versus* I-2 are represented as # and, *versus* IIA are represented as ¤. Measurement of Mean Grey Value does not allow differentiating IMTG content in the 4 fiber types. A more detailed analysis of IMTG stores, as lipid droplets’ density and size (D), allows for the differentiation of 4 metabolically distinct fiber types. Two populations of type I muscle fibers, I-1 and I-2, can be differentiated by muscle fiber crossectional area (E) and lipid droplet density. Type I-2 muscle fibers are similar in fiber crossectional area and lipid content and distribution to type IIA. Quantification of lipid and glycogen content in 10 human *VL* muscles before (black bars) and after exhausting exercise (grey bars) was performed. Glycogen content is expressed as mean grey value (F), lipid droplets’ density as number of lipid droplets in 3.6 µm^2^ (G) and, lipid droplets’ size as number of pixels (H) (Pixel size is 3.6*10^−3^ µm^2^). Two-way repeated Measures Analysis of Variance was performed and statistically significant differences between fiber types (C and D: *vs I-1, #vs I-2) and, between basal and exhausted muscle (F–H: *p<0.05, **p<0.01 and ***p<0.005) are represented.

Following the above described optimized protocols for lipid and glycogen fluorescence staining, the content and intracellular distribution of lipid and glycogen stores were investigated in VL muscle from young healthy subjects. Consecutive cryosections from each biopsy were cut and mounted on three different glass slides, two cryosections per slide. Basal and exhausted VL biopsies from the same subject were stained the same day. The first slide was stained for IMTG, the second slide was used to co-stain myosin heavy chain (MHC) I and II for fiber typing, and the third slide was used for co-immunostaining of glycogen and laminin. In figure 3A, representative images of IMTG staining, glycogen and laminin co-immunostaining and MHC I and II co-immunostaining are shown. It is visually obvious that two metabolically distinct populations of muscle fibers expressing MHC I exist; I-1 fibers being smaller and with higher lipid droplet density and I-2 fibers been bigger and with lower lipid droplet’s density. Each muscle fiber was identified in each of the three stained muscle crossections so that fiber type crossectional area, glycogen and IMTG content was measured, as well as LD density and size. Consecutive sections were used to classify type I, type IIA and type IIX muscle fibers by myofibrillar ATPase staining as previously described [19] (figure 3B). Type I-1 and I-2 were visually classified. The results confirmed our visual observations, 4 types of muscle fibers were identified: I-1, I-2, IIA and IIX. MHC I expressing muscle fibers can be divided into two populations: I-1 fibers have a smaller crossectional area (p<0.001) (figure 3E), a higher density of lipid droplets (p<0.001) (figure 3D), which are more homogeneous in size, and a tendency to lower glycogen content compared to I-2 fibers (figure 3C). Furthermore, LDs’ density was significantly lower in type IIX muscle fibers compared with IIA (figure 3D).The percentage of the different fiber types in VL muscle were: 26±5% of type I-1, 35±5 of type I-2, 22±4 of type IIA and 16±5 of type IIX. The proportional contribution of the different fiber types to the cross sectional area of VL muscle was 23±5% for I-1, 38±6% for I-2, 24±4% for IIA and 14±4% for IIX. In order to investigate potential differences in utilization of muscle lipid and glycogen stores during exercise between the 4 different fiber types, staining of glycogen and lipid stores and, co-immunostaining of MHCI and MHCII were performed on cryosection from VL muscle before and after exhaustive exercise. Results are presented and show that exhaustive exercise of VL muscle results in glycogen utilization in type IIA and IIX fibers (figure 3F). Biochemical analysis of muscle glycogen content of the same samples was previously performed [11] and showed a 56% decrease in muscle glycogen content in response to exhaustive exercise. The histochemical results presented here, when corrected by the muscle fiber type composition to calculate muscle glycogen content, show a 33% decrease in muscle glycogen in response to exhaustive exercise. The difference in the amount of utilized glycogen could be explained by the time between extraction of the biopsy and freezing/fixing of the muscle. It is key to minimize the time between muscle extraction and fixation, however, even when the muscle biopsy is immersed into fixative immediately after the biopsy is taken, it takes some time for the fixative to diffuse into muscle fibers, while deeping the biopsy into liquid nitrogen results in fast freezing of the tissue. The results reported here are in agreement with previous studies [20], where glycogen utilization during exhausting running was detected as a 30–45% in whole muscle, compare to the 25–33% glycogen reduction shown here. Furthermore, exhaustive exercise also resulted in lipid depletion detected as a reduction in LDs density in type I-1 and I-2 fibers (figure 3G) and, as a decrease in LDs size exclusively in type I-1 fibers (figure 3H). Two-way repeated measures ANOVA analysis showed fiber type differences on the response to exercise for LD size (p = 0.003) and muscle glycogen content (p<0.001). Biochemical measurement of muscle TG showed no significant reduction in response to exhaustive exercise, being TG content 49±2 and 55±5 mmol/g dry weight in basal and exhausted muscle, respectively. These results are not surprising since it has been previously reported that the presence of a single adipocyte in fraction of the muscle biopsy used to measure TG will totally mask any changes in intramyocellular TG [21]. However, the results obtained by histochemical analysis are in agreement with previous studies that have shown significant IMTG use after 60–120 min of whole-body exercise at intensities between 50 and 70% VO_2_peak using Folch extraction of TG in muscle biopsies [22], [23], ORO staining [24] or ^1^H-magnetic resonance spectroscopy [25]. Muscle lipid and glycogen content had previously biochemically measured in the same samples (ref). Biochemical measurements of muscle triacylglycerides.

## Conclusions

For optimal preservation of both IMTG and glycogen stores, muscle cryosections should not be air dried. Any cycle of freezing/thawing should be avoided since it results in loss of both lipid droplets and glycogen particles (Figure 2) [4]. Bodipy is the fluorescent dye of choice to image IMTG. It has a narrower emission spectra compared to ORO and, it does not affect light transmission (figure 1D). It is important to acknowledge that none of the classically used dyes are specific for either IMTG or glycogen. Both ORO and Bodipy bind to neutral lipids in the LDs but also in other intracellular structures. Thus, when imaging Bodipy or ORO, one should perform a lambda stack to assess the optimal range of wave length to image IMTG and, perform and publish high resolution images that allow the authors and readers to assess the specificity of the collected fluorescent signal. To increase the specificity of the glycogen staining, we have used a monoclonal antibody against glycogen. However, PAS staining can also be used if consecutive cryosections are pre-incubated with diastase and the stained structures are subtracted from the total PAS stain. Here we report the existence of two metabolically distinctive type I muscle fibers. Further studies are needed in order to learn more about the metabolic and functional role of these two new distinct type I muscle fiber populations. However, we would like to highlight that the classic conception that states that type I muscle fibers have more lipid than type II muscle fibers is not always true. Type I-2 and IIA muscle fibers have the same lipid content.
